# Progressive membrane-binding mechanism of SARS-CoV-2 variant spike proteins

**DOI:** 10.1016/j.isci.2022.104722

**Published:** 2022-07-04

**Authors:** Michael Overduin, Troy A. Kervin, Anh Tran

**Affiliations:** 1 Department of Biochemistry, University of Alberta, Edmonton, AB, Canada

**Keywords:** Virology, Structural biology, Protein structure aspects

## Abstract

Membrane recognition by viral spike proteins is critical for infection. Here we show the host cell membrane-binding surfaces of severe acute respiratory syndrome coronavirus 2 (SARS-CoV-2) spike variants Alpha, Beta, Gamma, Delta, Epsilon, Kappa, and Omicron as well as SARS-CoV-1 and pangolin and bat relatives. They show increases in membrane binding propensities over time, with all spike head mutations in variants, and particularly BA.1, impacting the protein’s affinity to cell membranes. Comparison of hundreds of structures yields a progressive model of membrane docking in which spike protein trimers shift from initial perpendicular stances to increasingly tilted positions that draw viral particles alongside host cell membranes before optionally engaging angiotensin-converting enzyme 2 (ACE2) receptors. This culminates in the assembly of the symmetric fusion apparatus, with enhanced membrane interactions of variants explaining their unique cell fusion capacities and COVID-19 disease transmission rates.

## Highlights

• Betacoronaviruses including SARS-CoV-2 bind membranes via spike protein heads
• Membrane-binding propensities are enhanced in variants, particularly in Omicron BA.1
• Networks of perpendicular and tilted states of spikes facilitate host cell docking
• Cell entry can occur through ACE2-dependent and independent routes

## Graphical abstract

**Figure:**
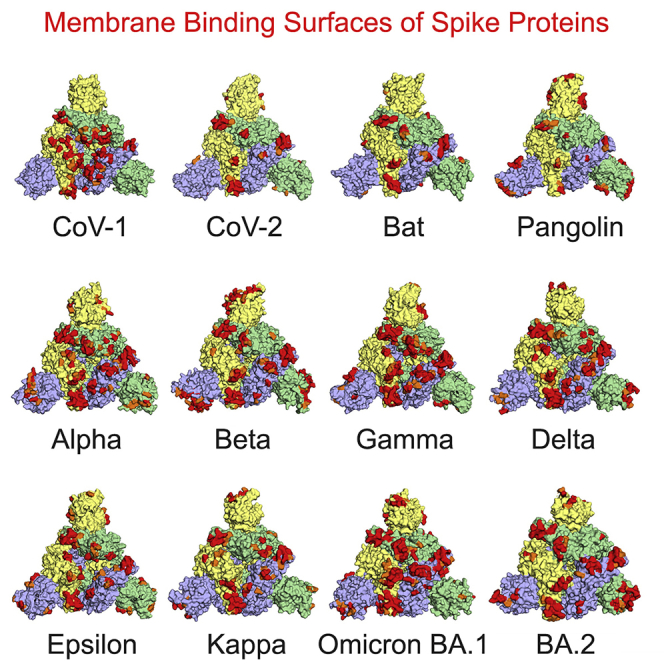

## Introduction

The COVID-19 pandemic is yielding SARS-CoV-2 variants of concern for which the evolutionary drivers require elaboration, as do sites targeted by therapeutic antibodies, vaccines, and inhibitors (Zhang et al., 2021b). The selection for increased fitness has yielded Omicron variants that transmit more rapidly and replicate faster and to higher levels in human bronchus where they primarily enter through the endosomal pathway (Hui et al., 2022). In contrast, the predecessors preferentially enter hosts by fusing with the plasma membranes deeper in the lung where the virus replicates more slowly, destroys respiratory tissues, and invades organs including the heart, kidney, brain, and liver, leading to cardiogenic shock, renal failure, neurological dysfunction, and lymphopenia (Satturwar et al., 2021). The entry and assembly processes involve viral particles binding to and crossing cellular and intracellular membranes, likely facilitated by the exposed spike protein (S).

The structure of the trimeric spike protein includes S1 (residues 14-685) and S2 (686-1273) subunits that are separated by a proteolytic cleavage site. The S1 subunit encompasses a signal peptide (SP), N-terminal domain (NTD), and receptor-binding domain (RBD) that projects away from the virus. The function of the NTD is unclear while the RBD recognizes the ACE2 glycoprotein through a receptor binding motif (RBM), thus mediating specific attachment to host cells. The S2 subunit includes a fusion peptide (FP) and heptapeptide repeat sequences 1 and 2 (HR1 and HR2), and anchors into the viral membrane through a transmembrane helix and palmitoylated cytoplasmic domain.

Mutations in the spike protein are thought to contribute to the increased transmissibility of SARS-CoV-2 variants of concern (Otto et al., 2021). The predominant explanation is that more transmissible variant spikes exhibit a higher affinity for the ACE2 receptor, although this does not necessarily account for the success of the Omicron variant (Ni et al., 2022; Pengcheng et al., 2022; Wu et al., 2022). Moreover, the Delta variant spikes induce enhanced cell-cell fusion even when ACE2 is expressed at minimal levels (Zhang et al., 2021c, 2022), whereas Omicron spikes mediate enhanced cell-to-cell transmission despite lower ACE2 affinity (Zeng et al., 2021). Accelerated cell-to-cell transmission of SARS-CoV-2 allows efficient spreading of the virus within the host in a manner that does not require ACE2 (Zeng et al., 2022). Consistent with this idea, there is growing evidence for ACE2-independent entry of SARS-CoV-2 into astrocytes (Andrews et al., 2021), neurons (Carossino et al., 2022), lung cells (Caccuri et al., 2021; Puray-Chavez et al., 2021), CHO-K1 cells (Liu et al., 2022), T-cells (Shen et al., 2022), immune cells (Ren et al., 2021), platelets and megakaryocytes (Zaid and Guessous, 2022). The isolated S1 subunit can cross the blood–brain barrier and is taken up by kidney, liver, and spleen without ACE2 involvement (Rhea et al., 2021). ACE2-independent interactions of viral particles that are stacked or in contact with intracellular membranes in human airway epithelium are visible by electron tomography (Pinto et al., 2022) and may contribute to intracellular virus entry, assembly, and trafficking. This involves fusion of small transport vesicles loaded with spike trimers with single membrane vesicles (Mendonça et al., 2021). Altogether this suggests that spike ectodomains directly engage phospholipid bilayers.

The absence of membranes from all atomic resolution structures of spike proteins necessitates computational approaches to understand such interactions. Membrane-binding sites can be predicted within protein structures using programs including Ez-3D (Schramm et al., 2012), Positioning of Proteins in Membranes (PPM; Lomize et al., 2012, 2022) and Membrane Optimal Docking Area (MODA; Kufareva et al., 2014). The latter program is freely available online and employs an algorithm that is knowledge-based, being trained to identify experimentally verified phospholipid-binding surfaces in 3D structures by assigning quantitative, lipid composition-independent membrane-binding propensity scores to each residue. We have used this approach to discover membrane recognition sites in bacterial and viral trafficking proteins (Bissig et al., 2013; Bryant et al., 2020), prions (Overduin et al., 2021b), and a large set of the many hundreds of eukaryotic membrane readers (Lenoir et al., 2015; Overduin and Kervin, 2021). Based on this experience we use MODA here to identify a series of membrane interaction sites and lipid bilayer poses of spike variant conformers, yielding a comprehensive mechanism of membrane binding.

## Results

### Spike heads contain conserved membrane-binding sites

Over 2,300 structures of spike protein subunits from various betacoronaviruses have been reported (Gowthaman et al., 2021), but none contain lipid bilayers, and how they bind host cell membranes remains unclear. However, there are spike structures complexed with biliverdin (Rosa et al., 2021) and fatty acids (Bangaru et al., 2020; Carrique et al., 2020; Toelzer et al., 2020; Yan et al., 2021) bound within the ectodomain, and these lipid molecules can reside in membranes. To identify the bilayer-interacting surfaces of each spike ectodomain, the membrane-binding propensities of >500,000 residues within 158 spike structures from SARS-CoV-1, 12 SARS-CoV-2 variants, as well as pangolin and bat homologs were measured using the MODA program (Bissig et al., 2013; Kufareva et al., 2014). The resulting model builds on our earlier mapping of the membrane-binding sites in wild-type SARS-CoV-2 spike trimers (Tran et al., 2022) and provides a unifying explanation for how betacoronaviruses interact with any lipid bilayer.

Existing structures and binding data support the direct interaction of spike ectodomains with lipid molecules. The S1 protein binds specifically to cholesterol via its RBD (Wei et al., 2020) and fatty acids via its NTD (Bangaru et al., 2020; Toelzer et al., 2020; Yan et al., 2021). The spike protein non-specifically extracts phospholipids independent of ACE2, thus directly permeabilizing membranes and inducing cytotoxicity in lung epithelial cells (Asandei et al., 2020; Luchini et al., 2021). Biliverdin binds the spike protein with an affinity of 9.8 nM (Rosa et al., 2021), and MODA analysis of lipid complexes reveals ligand contacts with residues exhibiting membrane-binding propensities including N99, I101, and M177, as well as I119, N121, and V126, which are adjacent to residues predicted to bind membranes including N120 and N125. The biliverdin complexes display enhanced membrane binding propensities in A123, N125, N343, T345, R346, V367, S371, A372, and S373, suggesting stabilization of membrane complexes by these residues. The detergent polysorbate 80 used in a Novavax vaccine also binds to an overlapping lipid-binding surface (Bangaru et al., 2020; Rosa et al., 2021), contacting residues including N99, N121, V126, as well as F175, which is also predicted by MODA to bind membranes in various spike structures. Closed complexes reveal that linoleic acid is bound within an interfacial pocket spanning adjacent RBD modules, and this is lined by R408 and residues in the Y369-K378 element that display elevated membrane-binding propensities in some RBD-up and ACE2-bound Omicron BA.1 structures. Interestingly these elements contribute to an allosteric trigger for membrane binding subject to the locking of the closed state by fatty acids (Oliveira et al., 2021). Hence various lipid ligands bind sites that overlap identifiable membrane docking surfaces within the spike trimer and may modulate bilayer binding. Moreover, the residues that engage both membranes and such ligand molecules are generally conserved (Figure 3), whereas variant mutations (Table 1) including Gamma R190S, Omicron S371F/L-S373P-S375F, and BA.4/5 R408S mutations are positioned to alter interactions with biliverdin or fatty acids as well as with membranes.

**Table 1:** Mutations in variant SARS-CoV-2 spike protein NTD and RBD modules

Variant	Membrane-binding positions	Beside a membrane-binding position in sequence	Close in space to a membrane binding residue
Alpha	ΔH69, ΔV70, ΔY144, N501Y		
Beta	L18F, D80A, D215G, R246I, E484K, N501Y	ΔL241, ΔL242, ΔA243, K417N	
Gamma	L18F, T20N, P26S, R190S, E484K, N501Y	K417 N/T	D138Y
Delta	T19R, E156G, ΔF157, ΔR158, T478K	G142D, L452R	
Epsilon		W152C, L452R	
Kappa	E154K, E484Q	G142D, L452R	
Omicron BA.1	ΔH69, ΔV70, ΔV143, ΔY144, ΔY145, ΔN211, L212I, ins214EPE, G339D, S371L, S373P, S375F, N440K, G446S, S477N, T478K, E484A, Q493R, G496S, Q498R, N501Y, Y505H	A67V, G142D, K417N	T95I
Omicron BA.2	T19I, L24S, ΔP25, ΔP26, A27S, V213G, G339D, S371F, S373P, S375F, R408S, N440K, S477N, T478K, E484A, Q493R, Q498R, N501Y, Y505H	G142D, T376A, D405N, K417N	
Omicron BA.3	ΔH69, ΔV70, ΔV143, ΔY144, ΔY145, ΔN211, L212I, G339D, S371F, S373P, S375F, D405N, N440K, G446S, S477N, T478K, E484A, Q493R, Q498R, N501Y, Y505H	A67V, G142D, K417N	T95I
Omicron BA.4	T19I, L24S, ΔP25, ΔP26, A27S, ΔH69, ΔV70, V213G, G339D, S371F, S373P, S375F, R408S, N440K, S477N, T478K, E484A, F486V, Q498R, N501Y, Y505H	G142D, T376A, D405N, K417N, L452R	
Omicron BA.5	T19I, L24S, ΔP25, ΔP26, A27S, ΔH69, ΔV70, V213G, G339D, S371F, S373P, S375F, R408S, N440K, S477N, T478K, E484A, F486V, Q498R, N501Y, Y505H	G142D, T376A, D405N, K417N, L452R	

Of the mutations identified in the NTD and RBM regions of these variants, most are in positions predicted by MODA to bind membranes directly, others are sequentially beside motifs predicted to bind membranes, and the remainder are spatially adjacent to residues that are predicted to bind membranes. The positions of these mutations are also shown in Figure 4, Figure 5, Figure 6, Figure 7, and mutations from BA.2, BA.3 (Desingu et al., 2022), BA.4 and BA.5 lineages (Yamasoba et al., 2022) of Omicron are also included here.

**Figure 1:**
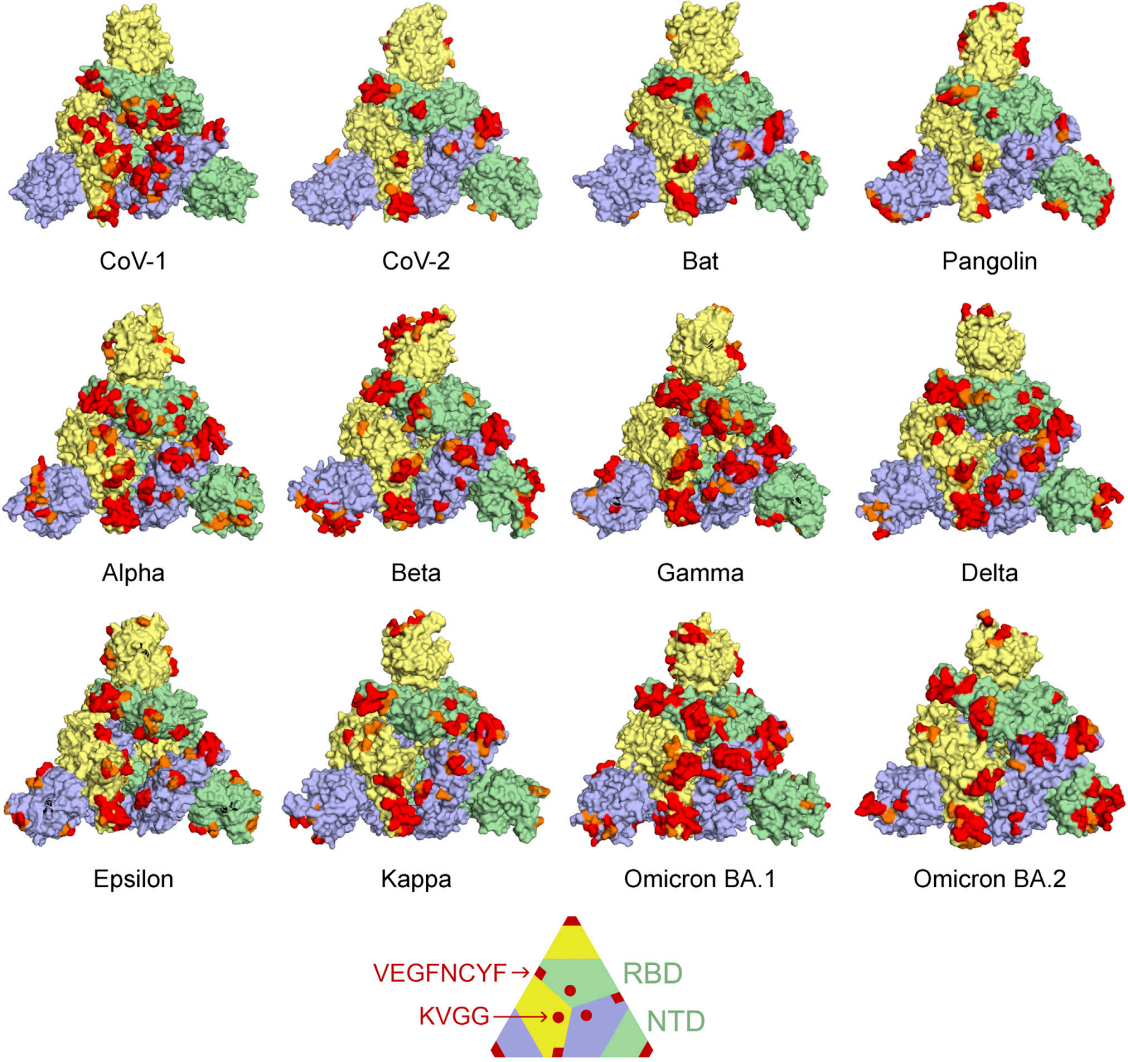
Membrane interaction sites in closed spike trimers of betacoronaviruses The view is taken from the perspective of a host cell. Surfaces with all RBDs positioned down are shown for the bat and pangolin coronaviruses, SARS CoV-1, SARS CoV-2, Alpha, Beta, Delta, Kappa, Omicron BA.1 and BA.2, Gamma, and Epsilon variants. Residues in orange and red have MODA scores of 20–40 and over 40, respectively, in PDB files 5x58, 6zge, 7cn4, 7cn8, 7n1u, 7n1t, 7v7n, 7v7d, 7wk2, 7ub0, and I-TASSER models. The cartoon shows the positions of the peripheral NTD site (red trapezoids) and inner and outer RBD sites (red circles and diamonds with labeled motifs) that are predicted to bind host membranes in the three subunits that are colored yellow, green, and lavender.

In addition to sites pinpointed by lipid complexes, another membrane-binding surface of all the trimeric spike structures is consistently formed by the RBM. This is the largest such surface, comprising 83.4 ± 9.4% of the total membrane-binding propensity of entire SARS-CoV-2 spike proteins, and as it projects furthest away from the viral particle, it would likely dominate host membrane interactions. Although the closed spike trimer cannot engage ACE2, it offers three roughly symmetric membrane interactive KVGG-447 elements within the central apex of its head (Figure 1). Through this central feature emanates a strong dipole moment that is universally exhibited by closed spike trimers (Table 2), focusing long-range electrostatic attractions onto the KVGG triplet. We propose that this distal tip of the highly populated, closed state of the ectodomain is well-positioned to attract and dock host cell membrane surfaces, whether extracellular or intracellular.

**Table 2:** Structural states of betacoronavirus and variant spike proteins

Virus	Variant	RBD Position	State	MODA (RBD)	S-Dipole	Ligand	Resolution	PDB	Reference
SARS-CoV-2	Alpha	3 down	1.0	6972	8154	–	3.14	7n1u	(Cai et al., 2021)
SARS-CoV-2	Alpha	3 down	1.0	5105	6369	–	3.22	7lws	(Gobeil et al., 2021b)
SARS-CoV-2	Alpha	1 up	1.1	3939	7088	–	3.12	7lwv	(Gobeil et al., 2021b)
SARS-CoV-2	Alpha	1 up	1.1	3437	7048	–	3.19	7lwt	(Gobeil et al., 2021b)
SARS-CoV-2	Alpha	1 up	1.1	7119	6296	–	3.20	7edf	(Yang et al., 2021b)
SARS-CoV-2	Alpha	1 up	1.1	4161	7170	–	3.20	7edg	(Yang et al., 2021b)
SARS-CoV-2	Alpha	1 up	1.1	4329	8097	–	3.21	7n1v	(Cai et al., 2021)
SARS-CoV-2	Alpha	1 up	1.1	4677	7068	–	3.22	7lwu	(Gobeil et al., 2021b)
SARS-CoV-2	Alpha	1 up	1.1	4292	8437	–	3.33	7n1w	(Cai et al., 2021)
SARS-CoV-2	Alpha	2 up	1.2	6311	6600	–	3.30	7edi	(Yang et al., 2021b)
SARS-CoV-2	Alpha	3 up	5	349	14718	3 ACE2	3.30	7edj	(Yang et al., 2021b)
SARS-CoV-2	Beta	3 down	1.0	6486	12,029	–	3.11	7n1t	(Cai et al., 2021)
SARS-CoV-2	Beta	3 down	1.0	5249	8692	–	3.57	7lym	(Gobeil et al., 2021b)
SARS-CoV-2	Beta	1 up	1.1	5706	12287	–	2.90	7n1q	(Cai et al., 2021)
SARS-CoV-2	Beta	1 up	1.1	4235	9119	–	3.32	7lyo	(Gobeil et al., 2021b)
SARS-CoV-2	Beta	1 up	1.1	4561	9070	–	3.32	7lyn	(Gobeil et al., 2021b)
SARS-CoV-2	Beta	1 up	1.1	4274	9231	–	3.34	7lyq	(Gobeil et al., 2021b)
SARS-CoV-2	Beta	1 up	1.1	7916	9724	–	3.20	7v76	(Yang et al., 2022)
SARS-CoV-2	Beta	1 up	1.1	7853	9757	–	3.40	7v8c	(Yang et al., 2022)
SARS-CoV-2	Beta	1 up	1.1	8440	10227	–	3.50	7vx1	(Wang et al., 2021c)
SARS-CoV-2	Beta	2 up	1.2	7894	9839	–	3.30	7v77	(Yang et al., 2022)
SARS-CoV-2	Gamma	3 down	1.0	6856	21272	–	–	I-TASSER	(Zheng et al., 2021)
SARS-CoV-2	Gamma	1 up	1.1	6425	10446	–	3.30	7v79	(Yang et al., 2022)
SARS-CoV-2	Gamma	1 up	1.1	7057	10411	–	3.40	7v78	(Yang et al., 2022)
SARS-CoV-2	Gamma	2 up	1.2	6807	10650	–	3.40	7v7a	(Yang et al., 2022)
SARS-CoV-2	Gamma	3 up	1.3	n.a.	n.a.	–	3.50	n.a.	(Yang et al., 2022)
SARS-CoV-2	Gamma	2 up	4.2	1836	7698	2 ACE2	3.20	7v81	(Yang et al., 2022)
SARS-CoV-2	Gamma	3 up	5	368	11098	3 ACE2	2.80	7v82	(Yang et al., 2022)
SARS-CoV-2	Gamma	3 up	5	193	10939	3 ACE2	2.80	7v83	(Yang et al., 2022)
SARS-CoV-2	Delta	3 down	1.0	6829	10023	–	2.90	7v7n	(Yang et al., 2022)
SARS-CoV-2	Delta	3 down	1.0	4610	10948	–	3.10	7sbk	(Zhang et al., 2021c)
SARS-CoV-2	Delta	3 down	1.0	5805	11522	–	3.40	7w94	(Wang et al., 2022)
SARS-CoV-2	Delta	1 up	1.1	n.a.	8346	–	2.25	7tey	(Saville et al., 2022)
SARS-CoV-2	Delta	1 up	1.1	3981	10466	–	2.80	7v7q	(Yang et al., 2022)
SARS-CoV-2	Delta	1 up	1.1	3916	10350	–	2.90	7v7p	(Yang et al., 2022)
SARS-CoV-2	Delta	1 up	1.1	4189	10324	–	2.90	7v7o	(Yang et al., 2022)
SARS-CoV-2	Delta	1 up	1.1	3859	10624	–	2.90	7v7r	(Yang et al., 2022)
SARS-CoV-2	Delta	1 up	1.1	4161	10597	–	3.00	7v7s	(Yang et al., 2022)
SARS-CoV-2	Delta	1 up	1.1	4282	11683	–	3.10	7w92	(Wang et al., 2022)
SARS-CoV-2	Delta	1 up	1.1	n.a	8623	–	3.16	7tov	(Gobeil et al., 2022)
SARS-CoV-2	Delta	1 up	1.1	5653	8185	–	3.24	7tou	(Gobeil et al., 2022)
SARS-CoV-2	Delta	1 up	1.1	3880	11407	–	3.40	7sbl	(Zhang et al., 2021c)
SARS-CoV-2	Delta	1 up	1.1	n.a.	8690	–	3.40	7tpf	(Gobeil et al., 2022)
SARS-CoV-2	Delta	1 up	1.1	n.a.	8724	–	3.40	7tp8	(Gobeil et al., 2022)
SARS-CoV-2	Delta	1 up	1.1	n.a.	8575	–	3.48	7tp7	(Gobeil et al., 2022)
SARS-CoV-2	Delta	1 up	1.1	n.a.	8626	–	3.48	7tp9	(Gobeil et al., 2022)
SARS-CoV-2	Delta	2 up	1.2	4124	10797	–	3.00	7v7t	(Yang et al., 2022)
SARS-CoV-2	Delta	2 up	1.2	3310	10880	–	3.10	7v7v	(Yang et al., 2022)
SARS-CoV-2	Delta	2 up	1.2	4141	10990	–	3.00	7v7u	(Yang et al., 2022)
SARS-CoV-2	Delta	1 up	3.1	n.a.	4559	1 ACE2	3.27	7tex	(Saville et al., 2022)
SARS-CoV-2	Delta	2 up	3.2	4381	5863	1 ACE2	3.40	7w9b	(Wang et al., 2022)
SARS-CoV-2	Delta	3 up	3.3	4470	6052	1 ACE2	3.20	7w9c	(Wang et al., 2022)
SARS-CoV-2	Delta	3 up	3.3	2886	5489	1 ACE2	3.40	7w99	(Wang et al., 2022)
SARS-CoV-2	Delta	2 up	4.2	954	8318	2 ACE2	3.30	7v88	(Yang et al., 2022)
SARS-CoV-2	Delta	3 up	5	33	11273	3 ACE2	2.70	7v8a	(Yang et al., 2022)
SARS-CoV-2	Delta	3 up	5	48	11342	3 ACE2	2.80	7v89	(Yang et al., 2022)
SARS-CoV-2	Epsilon	3 down	1.0	5369	20498	–	–	I-TASSER	(Zheng et al., 2021)
SARS-CoV-2	Kappa	3 down	1.0	5191	11970	–	3.00	7v7d	(Yang et al., 2022)
SARS-CoV-2	Kappa	3 down	1.0	7989	13648	–	3.10	7sbp	(Zhang et al., 2021c)
SARS-CoV-2	Kappa	1 up	1.1	n.a.	8513	–	2.25	7tf3	(Saville et al., 2022)
SARS-CoV-2	Kappa	1 up	1.1	4684	12251	–	2.90	7v7e	(Yang et al., 2022)
SARS-CoV-2	Kappa	1 up	1.1	4171	12146	–	2.90	7v7f	(Yang et al., 2022)
SARS-CoV-2	Kappa	1 up	1.1	5605	11621	–	3.20	7vxe	(Wang et al., 2021c)
SARS-CoV-2	Kappa	2 up	1.2	5710	12597	–	3.10	7v7g	(Yang et al., 2022)
SARS-CoV-2	Kappa	2 up	3.2	n.a.	3864	1 ACE2	3.02	7tf0	(Saville et al., 2022)
SARS-CoV-2	Kappa	2 up	4.2	651	6318	2 ACE2	3.30	7v85	(Yang et al., 2022)
SARS-CoV-2	Kappa	3 up	5	35	9798	3 ACE2	2.80	7v86	(Yang et al., 2022)
SARS-CoV-2	Omicron BA.1	3 down	1.0	9172	9758	–	2.56	7wp9	(Yin et al., 2022)
SARS-CoV-2	Omicron BA.1	down	1.0	n.a.	7853	–	2.79	7t9j	(Mannar et al., 2022)
SARS-CoV-2	Omicron BA.1	3 down	1.0	4836	10765	–	3.10	7wk2	(Hong et al., 2022)
SARS-CoV-2	Omicron BA.1	3 down	1.0	3520	11247	–	3.10	7tnw	(Zhang et al., 2022)
SARS-CoV-2	Omicron BA.1	3 down	1.0	8660	9548	–	3.36	7tf8	(Gobeil et al., 2022)
SARS-CoV-2	Omicron BA.1	3 down	1.0	8676	9460	–	3.50	7tl1	(Gobeil et al., 2022)
SARS-CoV-2	Omicron BA.1	1 up	1.1	9244	11403	–	3.00	7tgw	(Ye et al., 2022)
SARS-CoV-2	Omicron BA.1	1 up	1.1	5382	12246	–	3.02	7qo7	(Ni et al., 2022)
SARS-CoV-2	Omicron BA.1	1 up	1.1	11654	6923	–	3.11	7thk	(Cerutti et al., 2022)
SARS-CoV-2	Omicron BA.1	1 up	1.1	9475	10391	–	3.29	7tb4	(Wang et al., 2021b)
SARS-CoV-2	Omicron BA.1	1 up	1.1	5329	11097	–	3.40	7wk3	n.a.
SARS-CoV-2	Omicron BA.1	1 up	1.1	9587	12937	–	3.40	7wg6	(Cui et al., 2022)
SARS-CoV-2	Omicron BA.1	1 up	1.1	6242	11702	–	3.40	7to4	(Zhang et al., 2022)
SARS-CoV-2	Omicron BA.1	1 up	1.1	n.a.	n.a.	–	3.40	7tei	(Gobeil et al., 2022)
SARS-CoV-2	Omicron BA.1	1 up	1.1	6237	11215	–	3.40	7wvn	(Hong et al., 2022)
SARS-CoV-2	Omicron BA.1	1 up	1.1	3911	9486	–	3.41	7wvo	(Hong et al., 2022)
SARS-CoV-2	Omicron BA.1	1 up	1.1	9654	10175	–	3.50	7tl9	(Gobeil et al., 2022)
SARS-CoV-2	Omicron BA.1	1 up	3.1	1885	4158	1 ACE2	2.77	7wpa	(Yin et al., 2022)
SARS-CoV-2	Omicron BA.1	1 up	3.1	2440	2511	1 ACE2	2.90	7ws9	(Guo et al., 2022)
SARS-CoV-2	Omicron BA.1	1 up	3.1	3085	3906	1 ACE2	3.13	7xo5	(Xu et al., 2022)
SARS-CoV-2	Omicron BA.1	1 up	3.1	3423	4672	1 ACE2	3.69	7wk4	(Hong et al., 2022)
SARS-CoV-2	Omicron BA.1	2 up	3.2	5184	5256	1 ACE2	3.66	7wk5	(Hong et al., 2022)
SARS-CoV-2	Omicron BA.1	2 up	3.2	5261	5243	1 ACE2	3.70	7wvp	(Hong et al., 2022)
SARS-CoV-2	Omicron BA.1	3 up	3.3	7420	5770	1 ACE2	4.04	7wvq	(Hong et al., 2022)
SARS-CoV-2	Omicron BA.1	2 up	4.2	542	8309	2 ACE2	2.45	7t9k	(Mannar et al., 2022)
SARS-CoV-2	Omicron BA.1	2 up	4.2	1070	7679	2 ACE2	3.00	7ws8	(Guo et al., 2022)
SARS-CoV-2	Omicron BA.1	2 up	4.2	953	4278	2 ACE2	3.24	7xo4	(Xu et al., 2022)
SARS-CoV-2	Omicron BA.1	2 up	4.2	2523	9770	2 ACE2	3.30	7xid	(Xu et al., 2022)
SARS-CoV-2	Omicron BA.1	monomer	n.a.	9216	n.a.	–	–	Robetta	(Baek et al., 2021)
SARS-CoV-2	Omicron BA.2	3 down	1.0	6356	10173	–	3.31	7ub0	(Stalls et al., 2022)
SARS-CoV-2	Omicron BA.2	3 down	1.0	5908	9760	–	3.35	7ub5	(Stalls et al., 2022)
SARS-CoV-2	Omicron BA.2	3 down	1.0	6571	10146	–	3.52	7ub6	(Stalls et al., 2022)
SARS-CoV-2	Omicron BA.2	1 up	3.1	2537	3265	1 ACE2	3.20	7xoa	(Xu et al., 2022)
SARS-CoV-2	Omicron BA.2	2 up	4.2	511	4683	2 ACE2	3.30	7xob	(Xu et al., 2022)
SARS-CoV-2	Omicron BA.2	2 up	4.2	1011	7916	2 ACE2	3.38	7xo7	(Xu et al., 2022)
SARS-CoV-2	Omicron BA.2	3 up	5	619	12139	3 ACE2	3.48	7xo8	(Xu et al., 2022)
SARS-CoV-2	Omicron BA.2	monomer	n.a.	11796	n.a.	–	–	Robetta	(Baek et al., 2021)
SARS-CoV-2	Omicron BA.3	monomer	n.a.	10121	n.a.	–	–	Robetta	(Baek et al., 2021)
SARS-CoV-2	Omicron BA.4	monomer	n.a.	5180	n.a.	–	–	Robetta	(Baek et al., 2021)
SARS-CoV-2	Omicron BA.5	monomer	n.a.	5180	n.a.	–	–	Robetta	(Baek et al., 2021)
SARS-CoV-2	–	3 down	1.0	4678	7486	–	2.40	6xlu	(Zhou et al., 2020)
SARS-CoV-2	–	3 down	1.0	3341	7377	–	2.50	7jwy	(Zhou et al., 2020)
SARS-CoV-2	–	3 down	1.0	3928	9583	–	2.60	6zge	(Wrobel et al., 2020)
SARS-CoV-2	–	3 down	1.0	2384	9255	–	2.70	7df3	(Xu et al., 2021)
SARS-CoV-2	–	3 down	1.0	5017	6980	–	2.80	6vxx	(Walls et al., 2020)
SARS-CoV-2	–	3 down	1.0	4538	7459	–	2.80	7kdk	(Gobeil et al., 2021a)
SARS-CoV-2	–	3 down	1.0	n.a.	7675	–	2.83	7tlc	(Gobeil et al., 2022)
SARS-CoV-2	–	3 down	1.0	n.a.	7619	–	2.89	7tld	(Gobeil et al., 2022)
SARS-CoV-2	–	3 down	1.0	n.a.	7737	–	3.06	7tlb	(Gobeil et al., 2022)
SARS-CoV-2	–	3 down	1.0	n.a.	7667	–	3.13	7tla	(Gobeil et al., 2022)
SARS-CoV-2	–	3 down	1.0	n.a.	4396	–	2.90	6x79	(McCallum et al., 2020)
SARS-CoV-2	–	3 down	1.0	3522	9695	–	2.90	6xr8	(Cai et al., 2020)
SARS-CoV-2	–	3 down	1.0	3866	9624	–	2.90	6zgi	(Wrobel et al., 2020)
SARS-CoV-2	–	3 down	1.0	2694	9430	–	3.00	7ddd	(Zhang et al., 2021a)
SARS-CoV-2	–	3 down	1.0	n.a.	7808	–	3.00	6zow	(Melero et al., 2020)
SARS-CoV-2	–	3 down	1.0	3197	7711	–	3.01	7kdg	(Gobeil et al., 2021a)
SARS-CoV-2	–	3 down	1.0	6960	8057	–	3.10	6xm5	(Zhou et al., 2020)
SARS-CoV-2	–	3 down	1.0	2094	7223	–	3.22	6x6p	(Herrera et al., 2021)
SARS-CoV-2	–	3 down	1.0	4904	9607	Biliverdin	2.85	7nta	(Rosa et al., 2021)
SARS-CoV-2	–	3 down	1.0	4229	9007	Biliverdin	3.60	7nt9	(Rosa et al., 2021)
SARS-CoV-2	–	3 down	1.0	2893	6693	Linoleic acid	2.27	7qur	n.a.
SARS-CoV-2	–	3 down	1.0	2658	9491	Linoleic acid	2.70	7dwy	(Yan et al., 2021)
SARS-CoV-2	–	3 down	1.0	2823	6982	Linoleic acid	2.85	6zb5	(Toelzer et al., 2020)
SARS-CoV-2	–	3 down	1.0	3915	7050	Linoleic acid	3.03	6zb4	(Toelzer et al., 2020)
SARS-CoV-2	–	3 down	1.0	2750	9644	Linoleic acid	3.60	7jji	(Bangaru et al., 2020)
SARS-CoV-2	–	3 down	1.0	2716	8618	Steric acid	3.10	7z3z	(Carrique et al., 2020)
SARS-CoV-2	–	1 up	1.1	3085	8592	–	2.50	6xm3	(Zhou et al., 2020)
SARS-CoV-2	–	1 up	1.1	3,225	8231	–	2.50	6xm4	(Zhou et al., 2020)
SARS-CoV-2	–	1 up	1.1	2845	8022	–	2.70	6xm0	(Zhou et al., 2020)
SARS-CoV-2	–	1 up	1.1	2577	7887	–	3.10	6zp5	(Melero et al., 2020)
SARS-CoV-2	–	1 up	1.1	6205	8574	–	3.30	7dwz	(Yan et al., 2021)
SARS-CoV-2	–	1 up	1.1	7378	7918	–	3.50	7eaz	(Yang et al., 2021a)
SARS-CoV-2	–	1 up	1.1	346	8675	–	3.10	7dx1	(Yan et al., 2021)
SARS-CoV-2	–	1 up	1.1	382	8416	–	3.20	6vyb	(Walls et al., 2020)
SARS-CoV-2	–	1 up	1.1	n.a.	7760	–	3.30	6zp7	(Melero et al., 2020)
SARS-CoV-2	–	1 up	1.1	4128	7480	–	3.40	6z97	(Huo et al., 2020)
SARS-CoV-2	–	1 up	1.1	1869	6883	–	3.46	6vsb	(Wrapp et al., 2020)
SARS-CoV-2	–	3 up	3.3	301	4868	1 ACE2	3.00	7dx6	(Yan et al., 2021)
SARS-CoV-2	–	3 up	3.3	214	4535	1 ACE2	3.30	7dx5	(Yan et al., 2021)
SARS-CoV-2	–	3 up	3.3	1966	4349	1 ACE2	3.85	7kne	(Zhou et al., 2020)
SARS-CoV-2	–	3 up	4.3	1129	8495	2 ACE2	3.74	7knh	(Zhou et al., 2020)
SARS-CoV-2	–	3 up	4.3	1301	8519	2 ACE2	3.62	7kmz	(Zhou et al., 2020)
SARS-CoV-2	–	3 up	5	476	12087	3 ACE2	3.91	7kni	(Zhou et al., 2020)
SARS-CoV-2	–	3 up	5	291	11911	3 ACE2	3.64	7kms	(Zhou et al., 2020)
SARS-CoV-1	–	3 down	1.0	3368	1689	–	3.20	5x58	(Yuan et al., 2017)
SARS-CoV-1	–	3 down	1.0	3910	3496	–	3.80	5xlr	(Gui et al., 2017)
SARS-CoV-1	–	1 up	1.1	3751	3073	–	3.30	6crz	(Kirchdoerfer et al., 2018)
Pangolin CoV	–	3 down	1.0	607	7429	–	2.50	7cn8	(Zhang et al., 2021d)
Pangolin CoV	–	3 down	1.0	3688	7205	–	2.90	7bbh	(Wrobel et al., 2021)
Bat CoV	RaTG13	3 down	1.0	1494	4745	–	2.93	7cn4	(Zhang et al., 2021d)
Bat CoV	RaTG13	3 down	1.0	1379	6851	–	3.10	6zgf	(Wrobel et al., 2020)

Only the higher resolution structures analyzed in this study are listed, along with the orientation of the RBD modules, conformational state (as depicted in Figure 8), dipole moment (Debyes), names and numbers of bound ligand molecules, resolution (Å), PDB code, and reference. Where information is not available such as in the case of a missing PDB file or a lack of substantial structure, “n.a.” is shown.

**Figure 2:**
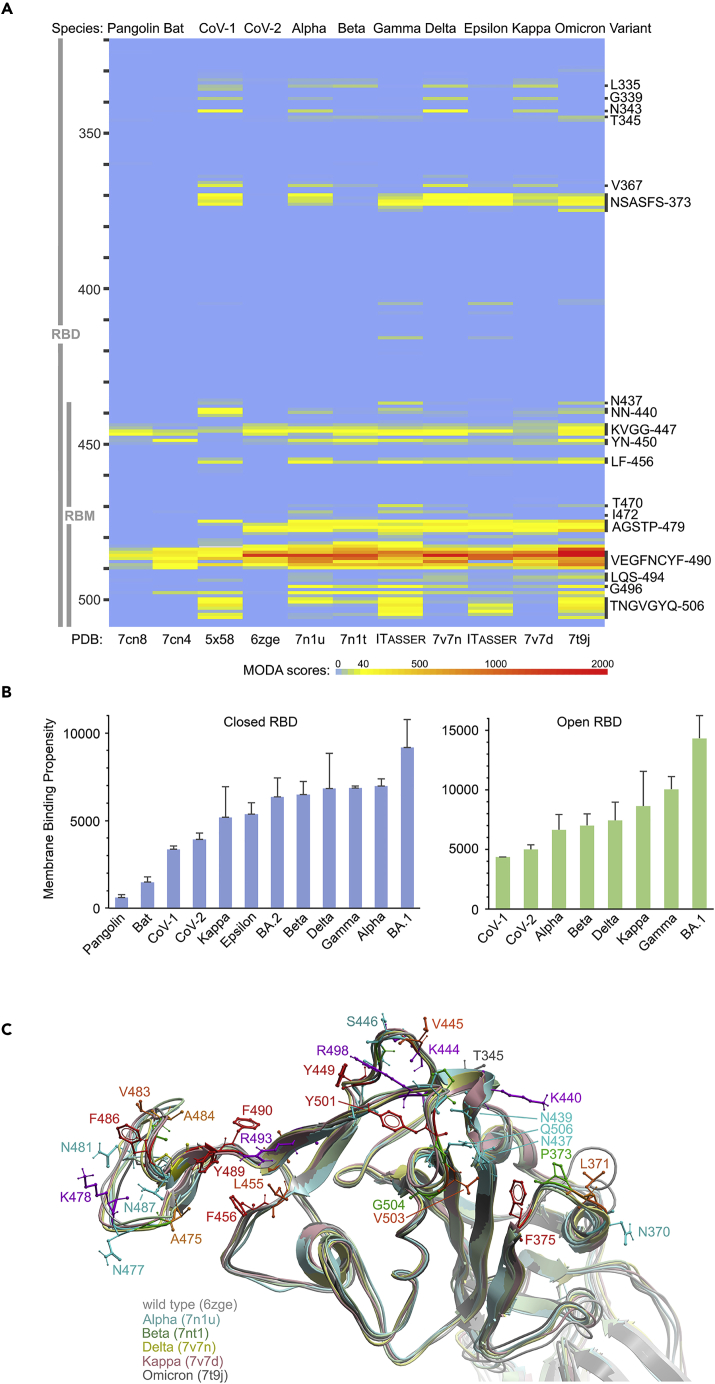
Betacoronavirus and variant spike membrane-binding elements (A) The membrane-binding propensities of pangolin, bat, SARS-CoV-1, CoV-2 wild-type as well as of cariant CoV-2 Alpha, Beta, Gamma, Delta, Epsilon, Kappa, and Omicron BA.1 spike protein residues are shown. Motifs that have substantial MODA scores are labeled on the right using the founder SARS-CoV-2 sequence. Positions are colored light blue–yellow–red to indicate MODA scores from 0 to 40 to 2000 as in the lower right scale. (B) The total MODA scores of residues in membrane-binding motifs in the single-up and all-down RBDs are shown in an increasing order. The closed trimer scores are for PDB files indicated in (A), except for Omicron BA.1 and BA.2 that are taken from 7wp9 and 7ub0, respectively. The open trimer scores are taken from the three highest resolution RBDs with fully resolved structures. (C) The RBD structures from wild type SARS-CoV-2 and five variants shown in (A) are superimposed and color-coded. The sidechains of Omicron BA.1 residues with MODA scores over 20 are shown to depict its major membrane-binding surface.

The central KVGG motif’s membrane docking propensity is especially variable, exhibiting 2-fold higher MODA scores in the Omicron BA.1 and BA.3 variants, where it is mutated to KVSG (Figure 2A). This central element is surrounded by a set of AGSTP-479 motifs that also exhibit the highest membrane-binding propensities in SARS-CoV-2 variants of concern, especially in individual subunits of Omicron structures that present an atypical AGNKP motif here, whereas the corresponding bat, pangolin, and SARS-CoV-1 elements exhibit no such propensity in their corresponding elements. Similarly, the proximal VEGFNCYF-490 motif exhibits the highest membrane-binding propensities in SARS-CoV-2 variants, especially in Omicron structures, which contain an E484A mutation. This eliminates a negative charge that would otherwise repel anionic lipid bilayer surfaces. Thus, the closed states of spike trimers are likely to mediate the initial docking to host cell membrane surfaces through this variable and expansive set of exposed RBM elements. We propose that this allows viral particles to engage host cells before forming specific receptor complexes, thereby localizing and orienting the multiple spike trimers before fusion events.

### Membrane interactivity in variants of concern

**Figure 3:**
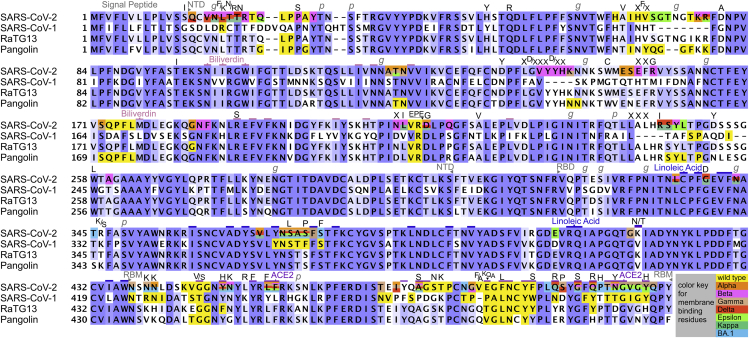
Alignment of betacoronavirus spike protein sequences Membrane-binding residues are highlighted in yellow for closed structures of SARS-CoV-1, bat and pangolin spike, or SARS-CoV-2 and its variants, with the latter being color-coded as shown in the bottom right key. Glycosylated and phosphorylated residue positions are indicated with “*g*” and “*p*.” The NTD, RBD and RBM boundaries are indicated by gray arrows. Residues that are within 4.5 Å of a bound ACE2 molecule in PDB 6lzg or that contact linoleic acid or biliverdin are indicated with upper bars colored purple, blue, and lavender, respectively. SARS-CoV-2 variant mutations are indicated above the sequences in uppercase black letters, with deletions and multiple variant mutations denoted by “X” and “/,” respectively, and an “EPE” insertion with an inverted triangle.

The fitness of coronaviruses is evolving over time, leading to enhanced host cell affinity and antibody evasion. When compared, the RBM’s membrane-binding propensities are ranked CoV-2 > CoV-1 > bat > pangolin (Figure 2B). This suggests greater lipid bilayer interactions, with the acquisition of KV-445 and VEGF-486 motifs by SARS-CoV-2 offering such gains of function (Figure 3). This progression continues with variants of concern gaining additional membrane-binding propensity owing to favorable substitutions. The density of interactive aromatic, aliphatic, basic, and polar groups in the spike head contributes to a broad membrane docking surface (Figure 2C) that is larger than the lipid recognition sites of any eukaryotic membrane reader (Overduin and Kervin, 2021). The overall structure of this array of binding motifs is conserved across betacoronaviruses, supporting a common membrane docking mode that appears to be growing over time. This trend culminates in Omicron BA.1, which exhibits the highest membrane-binding propensities within its RBD modules in both closed and open states (Figure 2B). Interestingly, all the positions that are mutated in variant NTD and RBD regions either exhibit membrane-binding propensity themselves or are sequentially or structurally next to another residue that does (Table 1). This suggests that membrane-binding is a widespread driver of viral fitness, in addition to other well-known factors including the ratio of open and closed states and the efficiency of proteolytic cleavage of spike proteins.

### Spike tethering to membranes occurs in stages

**Figure 4:**
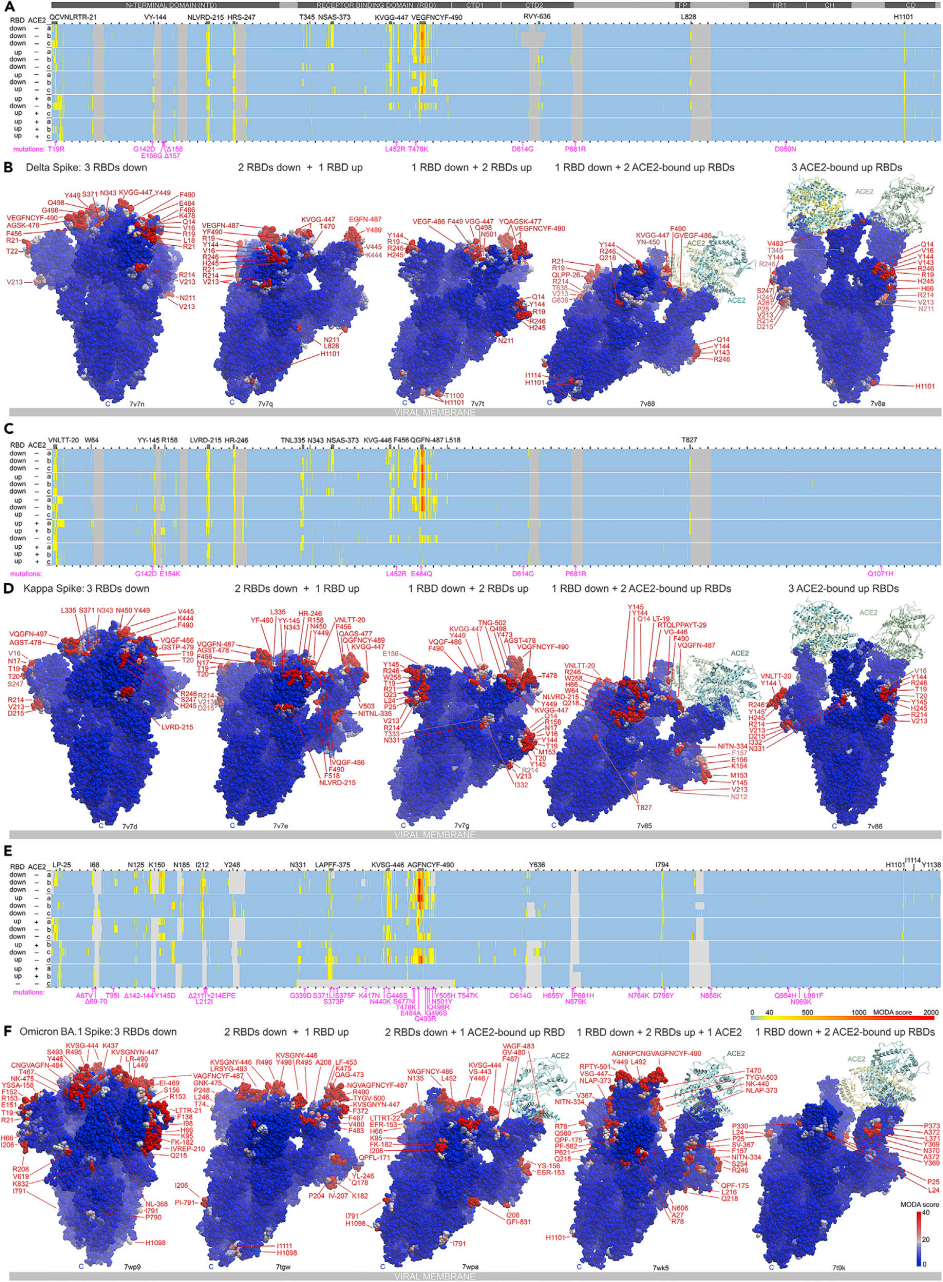
Membrane-binding poses of variant spike protein trimers The heatmaps show the membrane binding propensity of the residues in the Delta (A), Kappa (C), and Omicron BA.1 (E) spike trimer structures. RBD position and ACE2 occupancy are labeled left of the maps. Key motifs and mutations are labeled above and below, respectively. Positions are colored light blue–yellow–red to indicate MODA scores from 0 to 40 to 2000 as in the lower right scale, whereas gray indicates missing positions in the PDB files. The positions of the NTD, RBD, C-terminal domains (CTD) 1 and 2, fusion peptide (FP), heptad repeat 1 (HR1), central helix region (CH), and connector domain (CD) are shown above. The indicated conformational states are shown for the Delta (B), Kappa (D), and Omicron BA.1 (F) spike protein structures using PDBs listed above the viral membrane (gray slabs). The spike protein is tilted to position host membrane-binding interfaces above. Residues are labeled and colored pink-red based on MODA scores of 20– to 40+. The C-termini (C) connect to the viral membrane.

**Figure 5:**
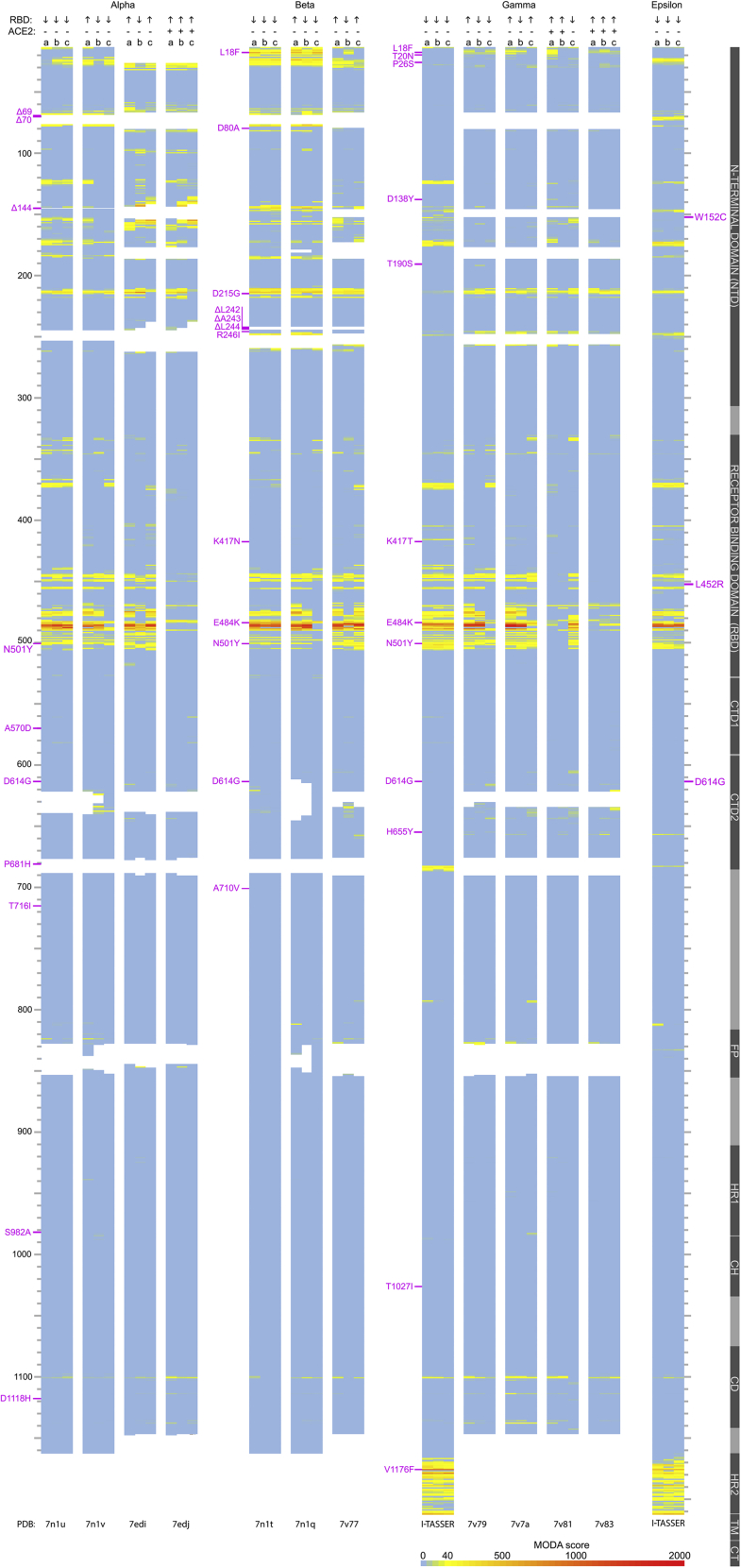
Membrane-binding propensities of Alpha, Beta, Gamma, and Epsilon SARS-CoV-2 variant spike protein structures Positions are colored light blue–yellow–red to indicate MODA scores from 0 to 40 to 2000, as indicated in the lower right scale, with residues missing in the indicated PDB files indicated in white within each vertical column. The up or down RBD positions (up or down arrows) and the absence or presence of ACE2 (minus or plus signs) are shown above the heatmap for the a, b, and c subunits of each trimer. The residue numbering is indicated on the left side along with the mutations found in the variants. The domain positions are shown on the right. The PDB entry names are shown below, with the Epsilon and Gamma closed spike ectodomain trimers modeled using I-TASSER (Zheng et al., 2021).

**Figure 6:**
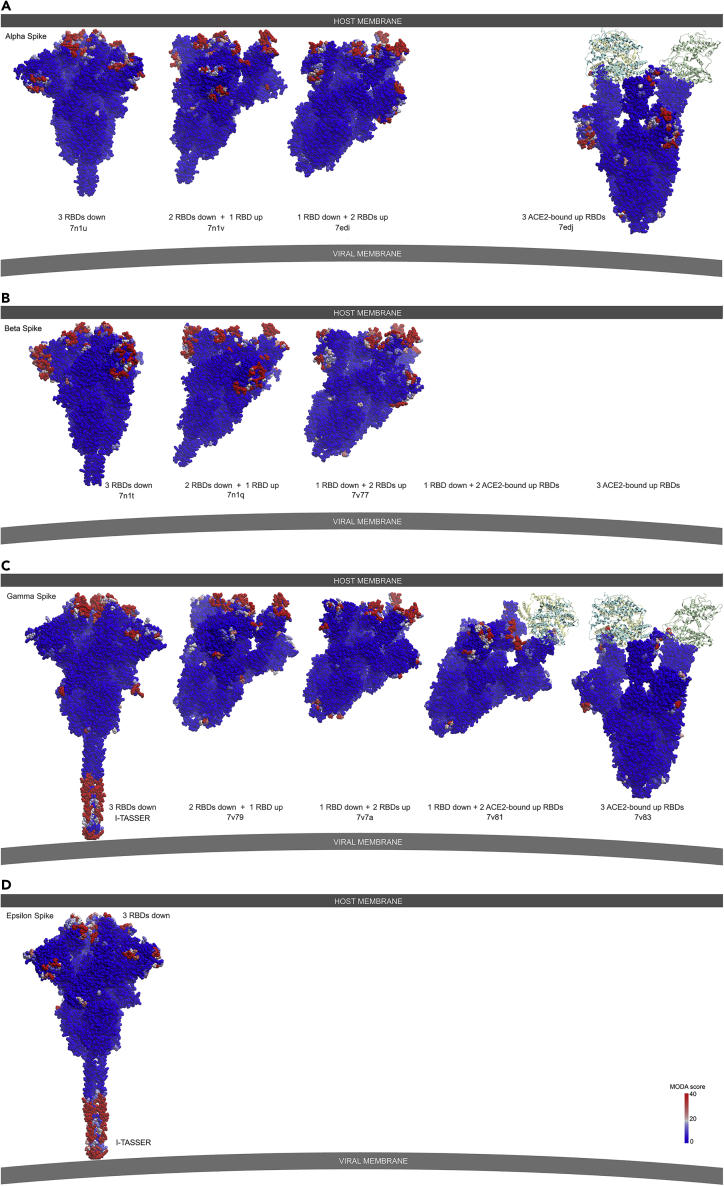
Series of membrane-binding poses of Alpha, Beta, Gamma, and Epsilon variant spike structures States of closed, one RBD up, two RBD up, and doubly or triply bound ACE2 molecules (ribbons) are shown, where available, for the trimeric Alpha (A), Beta (B), Gamma (C), and Epsilon (D) spike structures. The relevant PDB entry codes are labeled below. The host cell membrane and viral membrane are drawn as flat and curved gray slabs, respectively. The spike trimers are oriented to place the major membrane-binding surface near the host membrane and the C-termini toward the viral membrane. Residues are colored blue–pink–red on the surface depiction based on MODA scores of 0 to 20 to 40 + as in the lower scale.

Viruses dock to host cells through multiple steps of membrane association that can be seen in the array of variant spike structures. MODA analysis of a diverse range of high-resolution spike trimer structures (Table 2) yields a model with typically at least five major stages:

(1) First the closed trimer projects all three RBMs toward the host cell membrane like a blunt arrowhead, allowing all subunits to engage membranes by RBM residues scored as binders by MODA. This requires a perpendicular orientation to the membrane plane (Ke et al., 2020), with the convex docking surface being able to penetrate partway into the bilayer, potentially inducing curvature and fusigenicity (Zhang et al., 2021c). Indeed, complementary analysis of the symmetric membrane binding by the closed spike trimer (e.g. PDB: 7df3) using the PPM 3.0 server (Lomize et al., 2022) predicts bilayer penetration by V445 and G446 while large electrostatic dipoles (Table 2) could steer docking onto membrane surfaces.
(2) Secondly, a single RBD flips into the up position, allowing all three RBMs to form a large, flatter surface that docks obliquely to the host membrane when the trimer tilts ∼20° from perpendicular. The up subunit overshadows the third subunit’s RBM that is positioned clockwise to it, resulting in a slightly reduced MODA score that is 76.9 ± 15.6% that of the closed trimer.
(3) Another RBD then flips into the up position, providing an even larger flat surface that exhibits 123 ± 18.9% of the membrane-binding propensity of the single RBD up conformer. This and the preceding state with upturned RBDs are even more likely to engage membranes in Alpha, Beta, Gamma, Delta, and Kappa variants as they exhibit increased open state populations in various structures. This central intermediate state docks optimally to the host membrane via its RBM sites when the trimer is tilted by ∼30°. This more acute docking angle would draw the virus even closer to the host membrane.
(4) A pair of ACE2 molecules projecting from the host cell can then each engage one of the up subunits, displacing the host lipids from their RBM surfaces. This state has only 20.1 ± 8.0% of the membrane-binding propensity of the previous ACE2-free state. Its trimer axis is tilted by about 40°, allowing the remaining down RBM as well as NTD motifs to dock simultaneously to the host lipid bilayer while mediating an even closer contact with the viral membrane.
(5) When the remaining RBD flips up it can then bind to a third ACE2 molecule to form the symmetric, perpendicular complex that is poised to initiate membrane fusion. In this penultimate fusion state, the spike offers the lowest total RBD MODA score, at 10.1 ± 6.6% that of the previous complex. None the less, its N-terminal region elements including T22-A27 and V213-R214 still exhibit significant membrane-binding propensities. We suggest that these elements mediate S1 binding to the membrane once the cleaved spike protein dissociates, potentially assisting in fusion. This progressive model of host membrane binding appears generally conserved in Delta and Kappa spike series (Figure 4) as well as Alpha, Beta, Gamma, and Epsilon variants (Figures 5 and 6), although there are variations in the populations of constituent states as seen in cryo-electron microscopy (cryo-EM) studies, particularly in Omicron BA.1.

In addition to interactions with the host cell membranes, spikes also likely engage the viral membrane through peripheral interactions before fusion. The H1101 residue orients toward the viral membrane and exhibits membrane-binding propensity in all variants (Figures 4 and 6). Models of entire spike ectodomains available from I-TASSER (Zheng et al., 2021) consistently show not only 2.3 times higher dipole moments owing to the inclusion of heptad repeat (HR2) structural extension (Table 2) but also higher MODA scores from I1169-Q1201, even more so with Gamma’s V1176F spike mutation. We propose that this helical bundle promotes cell membrane attraction and can sit on the viral membrane when the spike trimer tilts, thus helping to absorb the shock of virus collision with a host cell.

**Figure 7:**
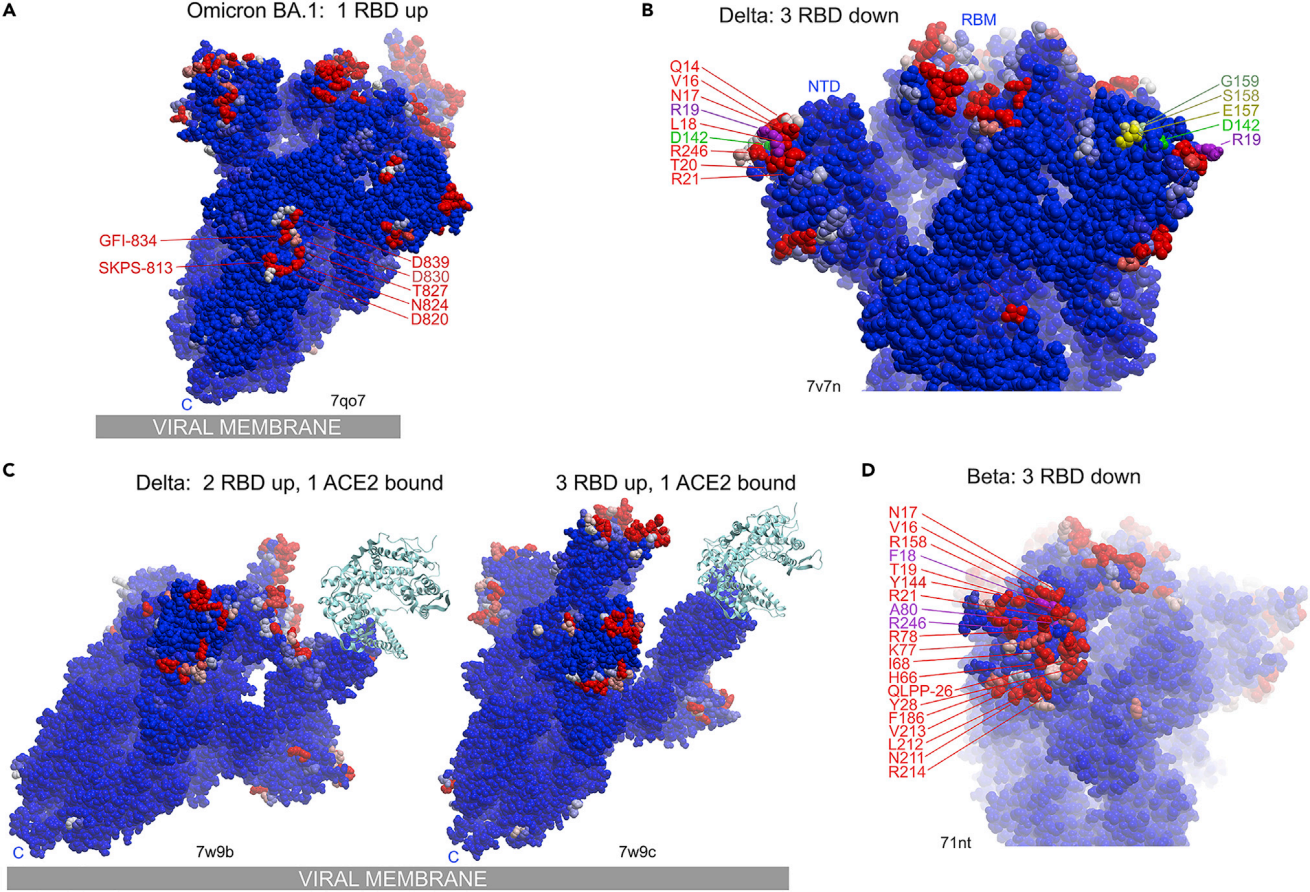
Structures of membrane-binding surfaces of SARS-CoV-2 variant spike trimers (A) The fusion peptide elements that unusually exhibit membrane-binding propensity in this Omicron BA.1 spike trimer structure are labeled. The blue surface depicts residues that are labeled and colored pink-red based on MODA scores of 20 to 40+. (B) membrane-binding surface on the outer edge of the closed Delta spike head. The T19R mutation in the Delta variant yields a membrane-binding NTD surface lined by Q14, V16-R21 and R246 that forms the top rim of the spike head. D142 influences the underlying β sheet and hairpin loop conformation and is near the position of deleted residues 157-158 and the E156G mutation. The latter residues precede the YHKNNKSWM sequence missing in this structure (PDB: 7v7n) but which displays significant MODA scores for Y145, H146, and K147, when present (PDB: 7w94). The membrane-interactive mutant residue R19 is in magenta and mutated D142, E157, S158, and G159 positions are color coded as shown. (C) Open Delta variant structures bound to ACE2 receptors (aqua ribbons) with additional RBD inflections. The images show two and three RBD modules positioned up on the left and right, respectively. The viral and host cell membranes would be below and above, respectively. (D) Membrane-binding surface on the rim of the Beta spike head. This largest continuous NTD membrane binding surface includes three mutated residues (magenta) as well as other residues exhibiting significant membrane-binding propensities (red) based on the MODA analysis.

Membrane fusion involves proteolysis and conformational changes that induce a helical wedge structure in the FP (Koppisetti et al., 2021), which is not generally predicted by MODA to engage membranes in the prefusion state. The primary exception is an open Omicron BA.1 structure (PDB: 7qo7c) where SKPS-813, D820, N824, T827, D830, GFI-834, and GD-839 are unusually accessible to the membrane (Figure 7A), suggesting that this variant conformer may be more fusion-ready. As the prefusion structures and MODA patterns are generally maintained, we suggest that a common mechanism of membrane docking can be used to evaluate the impacts of variant spike mutations.

### Membrane-binding modes by variants

**Figure 8:**
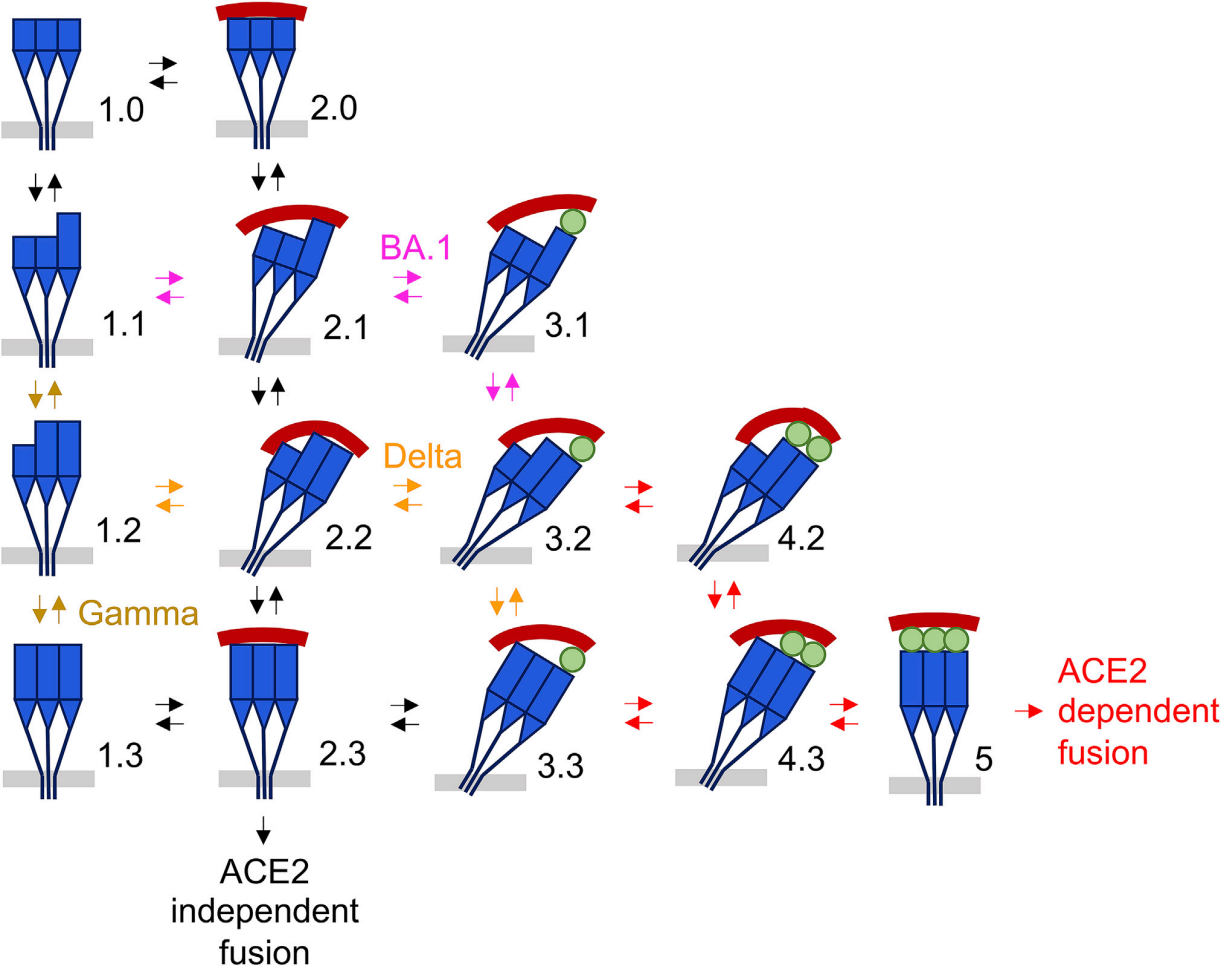
Progressive model of the spike:membrane attachment mechanism A single spike trimer is shown to depict the multiple states that populate the SARS-CoV-2 surface. The three unbound RBDs flicker between up and down states (tall and short blue rectangles, respectively) to yield interconverting spike states 1.0, 1.1, 1.2, and 1.3. Also drawn are the NTDs (blue triangles) and remainder of the S subunits (blue line) that span the viral membrane (light gray bar). The host cell membrane (dark red bars) is engaged in states 2 and above, and may lead to ACE2-independent fusion of viral particle and host cell membranes via the symmetric RBD-up state 2.3 (black arrows). The membrane-tethered spike trimer can bind a single ACE2 receptor (green circle) on the host cell surface either in state 3.1, 3.2, or 3.3, and then a second ACE2 molecule in state 4.2 or 4.3, and finally a third ACE2 molecule to form the canonical prefusion assembly state 5. Based on cryo-EM image distributions, the states that may be preferentially populated in Gamma, Delta, Omicron BA.1, or any variant are connected by gold, orange, magenta, and red arrows, respectively.

The abundant structures of the Delta variant spikes (Yang et al., 2022; Zhang et al., 2021c) provide explanations for how its mutations could influence membrane interactions. The T19R mutation introduces an exposed arginine that has the highest membrane-binding propensity in the closed Delta spike structure (PDB: 7v7n). Its sidechain points toward a host cell membrane and away from the virus surface, and is surrounded by predicted membrane-binding residues Q14, V16, N17, L18, T20, R21, and R246 (Figure 7B). Together they comprise the most basic and extensive lipid binding surface in the NTD and line the top rim of the spike trimer. This site appears best positioned to stabilize membrane interactions by tilted S trimers as well as the S1 subunit after its release following proteolytic cleavage. The G142D mutation sits next to this N-terminal membrane-binding element and alters the local conformation, whereas nearby mutations E156G, ΔF157, and ΔR158 shift the adjacent YYHK-147 element to be more accessible to the membrane. L452R and T478K introduce additional basic residues to the exposed surface of the RBM, increasing its overall propensity for host membranes (Figure 3B). Several structures suggest the presence of additional intermediates along the trajectory of membrane association. When only a single ACE2 molecule is bound by a Delta spike trimer (PDB: 7w99, 7w9b, 7w9c) one or two additional RBDs can flip up (Figure 7C). Although such states may be sparsely populated (Ke et al., 2020), the higher membrane-binding propensity offered by their free, upturned RBMs would promote host cell attachment, leaving the other RBD modules to flip between up and down positions. This would allow more sampling of the vicinity of the host cell membrane until ACE2 receptors are bound by all RBMs, thus providing more pathways (Figure 8) for convergence on the fusion-ready machine.

The series of Kappa spike structures (Wang et al., 2021c; Yang et al., 2022; Zhang et al., 2021c) exhibit membrane-binding propensities that mirror the patterns seen with Delta. For example, the G142D and L452R mutations found in Kappa have similar effects on the local structure and MODA scores. In contrast, the unique E154K substitution specifically elevates the local membrane-binding propensity in subunits with the NTD oriented toward the viral membrane (Figure 4D), with all residues in the MKSEFR-158 motif scoring as membrane binders. The E484Q mutation increases the membrane-binding propensity of this position 3.9-fold over the founder virus, leading to a 6.8% increase in the MODA score of the entire RBD. It is situated within Kappa’s QGFN-487 motif, which accounts for 61.6% of its overall MODA scores and 58.2% of the variability in its RBD scores, suggesting that this divergent element is essential for membrane binding. An even greater gain is seen with the Omicron E484A mutation, which leads to a 9.6-fold greater MODA score for both this position and the overall RBD membrane-binding propensity. The corresponding E484K mutations in Beta and Gamma variants provide 2.7- and 4.6-fold increases in their respective MODA scores. Hence each of these variant mutations involves exposed residues that promote membrane interactions.

The Alpha variant structures (Cai et al., 2021; Gobeil et al., 2022; Yang et al., 2021b) contain four mutations that occur in membrane-binding motifs. The N501Y mutation in the RBM increases the membrane-binding propensity at this position by 8.2-fold on average in the Alpha, Beta, Gamma, and Omicron variants. The deletion of H69 and V70 removes two residues from a loop that is disordered in all available experimental structures of the Alpha variant spike but displays membrane-binding propensity in complete I-TASSER models of the closed Gamma and Epsilon trimers (Figures 5 and 6). The Y144 deletion in the Alpha variant removes a residue next to a loop that is typically disordered but exhibits membrane-binding propensity in Beta spike structures (Cai et al., 2021; Gobeil et al., 2022; Wang et al., 2021c; Yang et al., 2022) where it is ordered.

The Beta variant contains an L18F mutation and exhibits the highest membrane-binding propensity at this position. This residue contributes to the NTD’s largest continuous membrane-binding motif and is located on the lateral surfaces of the spike head (Figure 7D). The Beta variant’s D80A mutation occurs next to this N-terminal motif as well as R78, a residue displaying membrane-binding propensity in variant structures including not only Beta but also Alpha, Delta, and Epsilon (Figure 5). The D215G mutation in the Beta variant leads to a 5.4-fold higher MODA score at this position and appears to elevate the membrane-binding propensity of the proximal NLVR-214 motif. The deletion of positions 241-243 as well as the R246I substitution alters this NTD interface that exhibits 4.5 times more total membrane-binding propensity here than the other variants.

Gamma variant spike structures (Yang et al., 2022) reveal positions of eight mutations that are predicted to alter membrane docking, and also appear to alter its populations of states (Figure 8). The K417 T/N mutation is next to G416 and F456, which likely mediate RBM-membrane interactions (Figures 5 and 6). The T20N mutation is situated next to the VNFT-19 motif, which consistently scores as a membrane binder in Gamma spike structures, whereas the P26S mutation alters the N-terminal backbone structure and is close to the predicted membrane-interactive VR-214 motif and Y28 sidechain. The partially buried R190S mutation appears to reduce the MODA scores of the preceding GNFK-187 motif. The D138Y mutation is also partially buried and removes stabilizing sidechain interactions with R21 and N81, thus altering the structure of the NTD membrane-binding element. The Gamma spike is unusual in its conformational distribution, which includes state 1.3 (Figure 8) with all three RBDs up, potentially maximizing membrane binding by the ACE2-free form (Yang et al., 2022).

The many mutations in Omicron BA.1 variant spikes affect both the membrane-binding features and populations, with a unique preference for state 3.1 (Figure 8), as evidenced by recent structures (Cerutti et al., 2022; Gobeil et al., 2022; Hong et al., 2022; Mannar et al., 2022; Ni et al., 2022; Wang et al., 2021b; Ye et al., 2022; Yin et al., 2022; Zhang et al., 2022). There are six mutations in the NTD and RBD that are also found in other variants, as well as 22 novel ones including A67V, which abuts membrane interactive H66 and I68. The T95I substitution alters the conformation of nearby K97, QGNF-186, and D215 residues that display membrane-binding propensities in Omicron BA.1 structures. The deletion of residues 142-144 alters the proximal loop structure such that the NKSW-152 gains the propensity to bind membranes. The combination of the N211 deletion, L212I substitution, and 214EPE insertion leads to higher MODA scores for the I212 position as compared with the Leu typically found here. The G339D mutation reduces favorable flexibility and alters the position of nearby N343, diminishing membrane-binding propensity often exhibited here in Delta and Kappa structures. The S371L, S373P, and S375F mutations alter the structure of this motif, increasing its membrane-binding propensity (Figures 4E and 4F). The N440K, T478K, Q493R, and Q498R substitutions add four positive charges to the membrane-binding surface and, along with G446S, S477N, G496S, and Y505H mutations, increase the membrane-binding score in the RBM surface 1.9-fold over other variants and 3.0-fold over the founder strain. This forms the most extensive membrane-binding surface on the spike trimer and may boost host cell membrane affinity by the Omicron BA.1 variant.

The Omicron BA.2 (Desingu et al., 2022), BA.4, and BA.5 lineages (Yamasoba et al., 2022) exhibit additional mutations including some that are unprecedented, like D405N and R408S (Table 1). These reduce the local membrane-binding propensity compared with BA.1 when the RBD elevates, contributing to an overall reduction in apparent membrane interactivity (Figure 2B). However, the G142D mutation found in BA.2 also increases the local membrane-binding propensity here. The VAGFNCYF-490 motif remains dominant, although F486V mutations in BA.4 and BA.5 reduce the membrane-binding propensity in these variants based on Robetta-derived models (Table 2). These alterations also affect ACE2 and antibody interactions, with other drivers such as immune system evasion remaining in play. Thus, each variant impacts the multiple functions of the spike protein, with membrane binding being one of several drivers that provides fertile ground for selecting favorable mutations.

### Post-translational modifications and antibodies influence membrane interactions

In contrast to the high mutation rates found in membrane-binding sites, other functional and regulatory sites are generally maintained in variants (Figure 3). Glycosylation of N17 and N343 sidechains could partially occlude the large membrane-binding surfaces presented by Beta, Delta, and Kappa variant spikes. Of the SARS-CoV-2 spike residues that are reportedly phosphorylated (Davidson et al., 2020) only T29 exhibits significant MODA scores in variants, suggesting a potential reduction of the local NTD-membrane interactions. However, the dominant membrane-binding surface within the RBD is free of such alterations and all variants presumably retain the ability to engage host membranes here even when metabolite-bound or post-translationally modified. The epitopes targeted by monoclonal antibodies (Cerutti et al., 2021; McCallum et al., 2021) used for treating COVID-19 disease (Ledford, 2021) overlap the membrane-binding sites of both founder and variant strains, indicating competitive interactions. Thus, the membrane interactions mediated by the RBM and NTD binding surfaces identified here provide explanations for the therapeutic effects of antibodies as they would interfere with the membrane engagement modes of the virus.

## Discussion

Profiling of variant spike structures in many conformational states can be used to construct a comprehensive model (Figure 8) for understanding how the many spikes on viral surfaces are employed during binding and entry to host cells (Jackson et al., 2021). The membrane is not visible in spike structures, but its binding surfaces are evident by MODA analysis. Based on this analysis we propose that these interfaces allow spike trimers on a virus to bind progressively to membranes during virus-cell fusion with or without ACE2 and during trafficking through endosomal and exocytic pathways. The tilted poses on lipid bilayers induced by the oblique binding surfaces may allow articulating spike trimers (Ke et al., 2020) to draw the viral membrane closer to the host membrane in preparation for a union while facilitating the engagement of additional proximal spikes. We suggest that subsequent formation of perpendicular prefusion spikes with three RBDs pointed up could drive dimples into the cell membrane to initiate fusion. Induction of a concave bilayer could also lead to the engagement of additional proximal spikes, thus enhancing the avidity of the contact zone. Such broad sandwiching of cells and viruses *via* multiple spikes may be essential for generating a fusion pore and gaining cell entry, as a single point of contact may be insufficient to complete this process. After connecting the viral particle to the host, the cleaved S1 subunits are released but could be retained locally as their membrane-binding sites interact with the lipid bilayer to assist in fusion, pore dilation, and penetration (Wang et al., 2021a) or alternatively, could penetrate the blood brain barrier or other organs to induce toxic effects (Petrovszki et al., 2022; Rhea et al., 2021). In addition to driving virus-cell fusion, spike trimers could contact membrane surfaces to facilitate virion assembly and stacking (Caldas et al., 2021; Eymieux et al., 2021; Klein et al., 2020; Mendonça et al., 2021; Pinto et al., 2022), and potentially facilitate membrane remodeling and cell-to-cell transmission even without expressed ACE2 receptors (Zeng et al., 2022). Such events may be enhanced by the larger membrane-binding surfaces of Omicron spikes (Zeng et al., 2021), demonstrating how variants navigate through custom pathways to gain entry. Greater membrane-binding propensity could also explain why the Delta spike is more fusogenic and increases entry into host cells when ACE2 is not expressed (Mlcochova et al., 2021), and why the Alpha spike alone accelerates cell-cell fusion and syncytium formation (Meng et al., 2021). However, much remains to be investigated, with these processes undoubtedly being influenced by factors such as the lipid composition as well as other host proteins such as tetherin, which prolongs the tethering of virions to cells (Perez-Caballero et al., 2009).

There are hints of specific lipids being recognized by spike proteins. Candidates involved in SARS-CoV-2 spike binding and viral entry include phosphatidylcholine, phosphatidylserine (Asandei et al., 2020; Luchini et al., 2021), phosphatidylinositol 3,5-bisphosphate (PtdIns (3,5)P_2_) (Kang et al., 2020; Ou et al., 2020), and cholesterol (Correa et al., 2021; Wei et al., 2020), which is known to be important for COVID-19 disease progression (Sanders et al., 2021). Spikes are also observed to co-locate with PtdIns(4,5)P_2_ in the plasma membrane (Raut et al., 2022). The interaction of linoleic acid within subunit interfaces stabilizes the closed conformation of the prefusion spike (Bangaru et al., 2020; Carrique et al., 2020; Toelzer et al., 2020; Yan et al., 2021), reducing the probability that upturned RBD conformers bind membranes via overlapping sites that are mutated in Omicron subvariants. The NTD module is also known to bind sialic acid on host cell surfaces (Krempl et al., 1997; Künkel and Herrler, 1993; Li et al., 2017; Park et al., 2019), while the RBD binds glycolipids in a manner that is critical for infection (Nguyen et al., 2021). Heparan sulfate proteoglycans also interact with the spike protein and enhance ACE2 binding (Clausen et al., 2020), with such cell surface recognition events enhancing infection rates and potentially influencing SARS-CoV-2 evolution (Shiliaev et al., 2021). Disruptive membrane interactions may contribute to the RBD’s independent ability to damage tissues when delivered intranasally (Kraus et al., 2022). Thus, there are a diverse array of previously identified phospholipid and glycolipid potential ligands and impacts to be explored, with the large membrane-binding surface of the spike head offering a multitude of sites that could bind and perturb membrane surfaces.

Preserving membrane-binding features could inform the development of vaccines. For example, those based on stabilized RBD trimers (Liang et al., 2022a, 2022b; Malladi et al., 2021; Routhu et al., 2021) could be designed to maintain the integrity of the spike’s dominant RBM membrane-binding surface for inducing potently neutralizing antibodies. While monomeric and dimeric RBD-based vaccines are also in development, these offer smaller membrane-binding surfaces that diverge from the native closed state. Vaccines that represent the intact trimeric form of the membrane-binding head can be optimized in light of a more complete mechanistic model. Therapeutic antibodies can now be designed in the knowledge that multivalent host membrane interactions by spike ensembles may limit accessibility while also serving as critical targets, with smaller nanobodies gaining easier access.

The lipid interactions of viruses can also be directly exploited for therapeutic benefit. Both phosphatidylglycerol (PG) and PtdIns lipids exhibit anti-viral effects against SARS-CoV-2, suppressing viral burden and preventing cytopathic effects in cellular models (Numata and Voelker, 2022). The use of lipids including PG for treating COVID-19 has been proposed (Bollag and Gonzales, 2020; Cattel et al., 2021; Ji et al., 2021; Veldhuizen et al., 2021), and clinical studies of administering phospholipid combinations for treatment of severe COVID-19 are showing improved patient outcomes (Bhatt et al., 2021; Busani et al., 2020; Dushianthan et al., 2020; Piva et al., 2021). This builds on earlier studies showing that PG and PtdIns lipids block the binding of virus particles to host cell plasma membranes and can be used to suppress transmission (Voelker and Numata, 2019). Similarly, the specific interaction of the respiratory syncytial virus with PG-based liposomes inhibits viral attachment to host cells, suppresses infection in mouse models (Numata et al., 2010), and prevents damage to the lung (Numata and Voelker, 2022). The influenza virus binds tightly to PtdIns and PG in a concentration-dependent manner, and these lipids inhibit virus attachment to host cells, reducing viral burden in mouse and ferret models of infection, preventing lung damage and promoting survival (Numata et al., 2020). Treatment with vesicles containing PG also specifically blocks vaccinia virus attachment to lung cells and protects against infection (Perino et al., 2011). Phospholipid-containing nanofibers have been proposed as anti-viral agents that bind to betacoronaviruses and thus block their entry into host cells (Mohamad et al., 2021). Structural insights into how lipids dock such as those presented here could inform the further development of more selective interventions.

The model presented here suggests that treatment with exogenous lipids may occlude the viral surfaces that would otherwise bind host cell membranes to initiate entry. A corollary is that definition of the ideal lipid composition for binding stably to viral surfaces would be desirable, with stable complementation of the basic binding surface of the RBD being recommended for specific intervention. Building on the development of phospholipid-based aerosol treatments, resilient nanoparticle-based treatments for COVID-19 are being designed to deliver stable lipid formulations (Kamat et al., 2021). Such agents could potentially not only maintain respiratory system function but also directly target a key step in infection. The design of vaccine adjuvants, which are often lipid-based, could also take advantage of the principles presented here to promote activity and stability (Mabrouk et al., 2022). Hence, the elucidation of how spike proteins bind membranes to initiate host contact opens many possibilities for targeting the specific pathways used for viral entry.

### Limitations of the study

The results presented here are based on a computational meta-study, and the membrane-binding sites and lipid bilayer poses identified here have not been experimentally validated by lipid binding assays or structural biology experiments. The multiple membrane-binding sites on several structural domains that rearrange within the trimers make it challenging for any one research group to experimentally validate these in the different variants within the time frame needed to inform the research community working to address the needs of the ongoing COVID-19 pandemic. Whereas the results from our MODA analysis were cross-validated using PPM, the latter provides more sparse data and is not optimized for analysis of multiprotein assemblies with an array of membrane-binding sites. Likewise, dipole moments, being consistent with the mechanism proposed here, predict long-range attractions that precede rather than equate with lipid bilayer binding. The network of spike conformers driving membrane association proposed here represents a simplified model, with many dynamic substates being differentially populated in variants with undefined kinetics and influences of engineered stabilizing mutations, expression methods, and structural biology preparations. Our current results are provided to inform the efforts of the research community looking to decipher the mechanism of the virus–host interaction, the effects of mutations, and the action of inhibitors, vaccines, adjuvants, and antibodies.

Our computational analysis focused on the available cryo-EM structures of spike ectodomains. Their resolutions were typically between 2.5 and 3.5 Å, and disordered loops particularly in the NTD were sometimes not visible. Hence, some sidechains, loops, and the transmembrane and cytoplasmic domains are not consistently defined, although these could influence membrane poses and lipid interactions. Moreover, the membrane-binding propensities of residues neighboring disordered loops and termini can be overpredicted by MODA owing to their greater apparent exposure to solvent in incomplete structures, and hence the MODA scores of such residues need to be treated with caution and should be cross-validated in other, more complete structures. Ideally, the complex structures of full-length proteins bound to lipid bilayers should be calculated using molecular dynamics simulations to generate more holistic models.

No phospholipids or bilayers are present in any of the spike structures yet are central to our proposed mechanism. The removal of biological lipids by the various detergents used to purify spike proteins could alter local binding site structures, although the overall structures are maintained. The conformations of the membrane-bound states have not been determined and could vary from the structures analyzed here. Ideally, the structures of the endogenous protein states bound to biological lipid bilayers should be resolved using native nanodiscs without destabilizing detergents, a feat that is becoming increasingly feasible with synthetic copolymers (Overduin et al., 2021a). The array of possible lipid ligands for the various membrane-binding surfaces of the spike structural states is unexplored and cannot be accurately predicted with current software and cannot be feasibly assayed within the time frame of this study. Moreover, the spike protein may recognize different lipid compositions, nanodomains, and curvatures as viral particles encounter different subcellular compartments. Some of these interactions may be individually weak and transient but become reinforced by the many spikes that can simultaneously engage the expansive, curved membrane surfaces of bound cells or vesicles. Although they are compatible, neither spike-spike nor cytoskeletal interactions have been specifically addressed here, and the viral membrane contains several other proteins that could also contribute to host membrane interactions. ACE2 molecules may not act alone to facilitate host cell surface recognition and can form multimers that mediate spike interactions. Other receptors may potentially be engaged by the virus on some cell types, whereas intracellular membrane recognition and cell-cell fusion interfaces may be devoid of such receptors. Hence, the spike function within cellular or *in vivo* settings is likely to be considerably more varied and complex than the model presented here, which is intended to provide a conceptual advance and framework for further studies.

## STAR★Methods

### Key resources table

**Table:** 

REAGENT or RESOURCE	SOURCE	IDENTIFIER
**Deposited data**		

CoV3D database	(Gowthaman et al., 2021)	Listing of spike protein structures
Structures of spikes	(Berman et al., 2000)	PDB entries 5x58, 5xlr, 6crz, 6vsb, 6vxx, 6vyb, 6x6p, 6x79, 6xlu, 6xm0, 6xm3, 6xm4, 6xm5, 6xr8, 6z97, 6zb4, 6zb5, 6zge, 6zgf, 6zgi, 6zow, 6zp5, 6zp7, 7bbh, 7cn4, 7cn8, 7ddd, 7df3, 7dwy, 7dwz, 7dx1, 7dx5, 7dx6, 7eaz, 7edf, 7edg, 7edi, 7edj, 7jji, 7jwy, 7kdg, 7kdk, 7kms, 7kmz, 7kne, 7knh, 7kni, 7lws, 7lwt, 7lwu, 7lwv, 7lym, 7lyn, 7lyo, 7lyq, 7n1q, 7n1t, 7n1u, 7n1v, 7n1w, 7nt9, 7nta, 7qo7, 7qur, 7sbk, 7sbl, 7sbp, 7t9j, 7t9k, 7tb4, 7tei, 7tex, 7tey, 7tf0, 7tf3, 7tf8, 7tgw, 7thk, 7tl1, 7tl9, 7tla, 7tlb, 7tlc, 7tld, 7tnw, 7to4, 7tou, 7tov, 7tp7, 7tp8, 7tp9, 7tpf, 7ub0, 7ub5, 7ub6, 7v76, 7v77, 7v78, 7v79, 7v7a, 7v7d, 7v7e, 7v7f, 7v7g, 7v7n, 7v7o, 7v7p, 7v7q, 7v7r, 7v7s, 7v7t, 7v7u, 7v7v, 7v81, 7v82, 7v83, 7v85, 7v86, 7v88, 7v89, 7v8a, 7v8c, 7vx1, 7vxe, 7w92, 7w94, 7w99, 7w9b, 7w9c, 7wg6, 7wk2, 7wk3, 7wk4, 7wk5, 7wp9, 7wpa, 7ws8, 7ws9, 7wvn, 7wvo, 7wvp, 7wvq, 7xid, 7xo4, 7xo5, 7xo7, 7xo8, 7xoa, 7xob, 7z3z
Uniprot database	(Uniprot Consortium, 2021)	Spike protein sequences QHR63300.2, A0A6G6A2Q2, P59594 and P0DTC2

**Software and algorithms**		

Clustal Omega program	(Sievers et al., 2011)	http://www.clustal.org/omega/
ICM Browser program	(Raush et al., 2009)	http://www.molsoft.com/icm_browser.html
I-TASSER program	(Zheng et al., 2021)	https://zhanglab.ccmb.med.umich.edu/I-TASSER/
Jalview 2 program	(Waterhouse et al., 2009)	https://www.jalview.org/
MODA program	(Kufareva et al., 2014)	https://molsoft.com/∼eugene/moda/modamain.cgi
PPM 3.0 program	(Lomize et al., 2022)	https://opm.phar.umich.edu/
Protein Dipole Moments Server	(Felder et al., 2007)	https://dipole.proteopedia.org/
Pymol program	(DeLano, 2014)	https://pymol.org/2/
Robetta program	(Baek et al., 2021)	https://robetta.bakerlab.org/

### Resource availability

#### Lead contact

Further information and requests for resources should be directed to and will be fulfilled by the lead contact, Michael Overduin (overduin@ualberta.ca).

#### Materials availability

This study did not generate new unique reagents.

### Method details

#### Sequence analysis

The sequences of spike proteins from Bat RaTG13 and Pangolin coronaviruses, SARS-CoV-1 and SARS-CoV-2 were obtained from UniProt entries QHR63300.2, A0A6G6A2Q2, P59594 and P0DTC2 (Uniprot Consortium, 2021). The sequences were aligned using with Clustal Omega (Sievers et al., 2011) and visualized using the Jalview 2 program (Waterhouse et al., 2009) to indicate sequence similarities. Mutations were identified from the literature and databases used therein(Uniprot Consortium, 2021) as were sites of phosphorylation (Bouhaddou et al., 2020; Davidson et al., 2020), glycosylation (Shajahan et al., 2020; Watanabe et al., 2020) and ligand binding including for biliverdin(Rosa et al., 2021), fatty acid (Bangaru et al., 2020; Carrique et al., 2020; Toelzer et al., 2020; Yan et al., 2021) and ACE2 receptor (Mannar et al., 2022; Yan et al., 2021; Yang et al., 2022; Zhou et al., 2020) within structures from the SARS-CoV-2 founder strain (D614) and variant spike proteins (Table 2).

#### Structures of spike proteins

Structures of spike protein trimers from four betacoronaviruses were obtained from CoV3D (Gowthaman et al., 2021), RCSB Protein DataBank (PDB) (Berman et al., 2000) and UniProt (Uniprot Consortium, 2021) sites and subsequent searches for coronavirus spike protein structures. High resolution structures of trimeric spike proteins, i.e. with resolutions of typically at least 3.5Å, were included in this study as sidechain conformations and surface residues become resolved in this range. Those that are most complete and have the highest resolution were selected as being most representative of a particular conformational state. Multiple spike structures of the same conformational state were included to increase confidence of conclusions regarding mutations, structural features and MODA scores. Those structures generated by the same team using similar protocols and constructs were compared to generate a stepwise trajectory of membrane association states where possible. The available structures of the various up and down conformational states of each RBD in a trimer as well as complexes state with one, two or three ACE2 receptor molecules were compared to ascertain how they were positioned to bind membrane surfaces in each case. Where experimental structural data was lacking for a closed state (i.e. Epsilon and Gamma variants), the 3D structure was obtained from I-TASSER (Zheng et al., 2021), and the Omicron subunit BA.2, BA.3, BA.4 and BA.5 S1 structures were modelled using RoseTTAFold’s deep-learning modelling method (Baek et al., 2021) to obtain the MODA scores of their residues.

#### Membrane binding propensities and sites

The MODA program was used to identify membrane binding surfaces in each spike trimer. This program assigns a membrane binding propensity to each residue in a structure based on the presence of features typically found in well-characterized protein structures known to bind lipid bilayers *via* exposed hydrophobic, aromatic and polar moieties as well as surface curvature and electrostatic properties (Kufareva et al., 2014). This approach has been previously used to discover lipid recognition surfaces on viral trafficking proteins (Bissig et al., 2013) as well as binding and regulatory features of many phosphoinositide recognition domains (Kervin and Overduin, 2021; Kervin et al., 2021; Overduin and Kervin, 2021). The spike structures were further analyzed with PyMol (DeLano, 2014) and the ICM Browser (Raush et al., 2009), which inputs ICB output files from the MODA program. MODA is unique in that it was designed to detect even novel membrane docking sites on multi-subunit assemblies based on trained using a library of peripheral membrane protein structures. Coordinates that are missing in experimental structures were not modelled, although other similar structures were considered where there were gaps. Sets of at least two residues that are adjacent in a sequence or structure and have MODA scores of at least 20 were considered to have significant membrane binding propensities, while such residues with MODA scores above 40 were predicted to have a substantial membrane binding propensities. The intensity of MODA scores of individual residues in membrane binding sites ranged up to 3000, which was taken to represent extremely high membrane propensity, while negative MODA scores were adjusted to zero. MODA scores were cross validated by comparison of their values in structures that were of the related sequence and conformational state. The CSV output files from MODA were analyzed in Excel to generate heatmaps and to assess whether there were significant differences in the positive scores of the various residues and sites. The average MODA scores of individual residues, motifs and domains and their standard deviations were calculated from each of the spike subunits. The RBD domain residues that display membrane binding propensities in multiple closed spike structures include L335, G339, N343, T345, V367, N370-S375, N437, N439, N440, K444-G447, Y449, N450, L455, F456, T470, I472, A475-P479, N481-F490, L492-S494, F496 and Q498-Q506 of the progenitor SARS-CoV2-2 sequence or the equivalent residue in the other spike sequences. Hence these residues were used to calculate the total MODA scores of the RBDs of the various spike structures, and are also color coded in the sequence alignment (Figure 3).

The other computational programs for prediction of membrane interacting surfaces of peripheral membrane proteins are Ez-3D (Schramm et al., 2012) and PPM (Lomize et al., 2012, 2022). The latter is available online to predict the spatial position of a protein structure on a fluid anisotropic solvent slab that represents a membrane-like environment and hence was used to validate the MODA results. We focussed on the highest resolution experimental structures of each variant trimeric spike protein in the down and one RBD up states as these offer more complete density for loops and sidechains. The PPM 3.0 program predicts 4.9 ± 5.01 membrane binding residues for these eleven spike structures (PDB; 7n1u, 7lwv, 7n1t, 7n1q, 7v79, 7v7n, 7tey, 7v7d, 7tf3, 7wp9 and 7tgw). In contrast MODA predicts 125.7 ± 36.8 membrane binding residues in these trimer structures. Of the membrane binding residues predicted by PPM 3.0, 74.1% (40 of 54) are also identified by MODA, which assigns all of them scores of 499.6 ± 819.8. The orientation of the open Alpha structure (PDB 7lwv) is an outlier, as it is predicted by PPM 3.0 to lie flat on the membrane rather than primarily by its RBM. When this outlier is removed, both programs agree on 91.4% (32 of 35) residues as membrane binders, with MODA consistently predicting membrane interactions mediated by the RBM surface. Thus, while the two methods generally identify the same membrane binding residues, PPM uses a less sensitive binary score while MODA predicts 25 times more interacting residues using a quantitative scale that yields more consistent binding modes for such multimeric structures.

### Quantification and statistical analysis

The membrane binding propensities of each residue in each subunit of the various spike trimer structures (Table 2) were determined using the MODA program (Kufareva et al., 2014). Based on this each residue’s average MODA score and standard deviation was calculated using Excel, as was the total MODA score of each structure’s RBD module including non-negative values within all membrane binding residues. To provide further evidence for membrane interactions, the dipole moments of spike trimers were calculated directly from their protein structures using the Protein Dipole Moments Server (Felder et al., 2007). Such dipoles are commonly found in phospholipid recognition domains and steer them onto membrane surfaces through long-range electrostatic forces (Lumb and Sansom, 2012). A linear regression test was performed to calculate whether the dipole moments of closed spike protein structures are related with the MODA scores of their triple RBD modules (Table 2). This shows a significant relationship [*F* (1, 41) = 9.65, P = 0.00352, *R*^*2*^ = 0.198], indicating that dipole-based steering and membrane binding of spike proteins are related.

## Data Availability

This paper analyzes existing, publicly available data. These accession numbers for the datasets are listed in the key resources table. This paper does not report original code. Any additional information required to reanalyze the data reported in this paper is available from the lead contact upon request.
